# Catch and Release: rare cell analysis from a functionalised medical wire

**DOI:** 10.1038/srep43424

**Published:** 2017-02-24

**Authors:** Shukun Chen, Amin El-Heliebi, Gerlinde Tauber, Tanja Langsenlehner, Michaela Pötscher, Karl Kashofer, Zbigniew T. Czyż, Bernhard Polzer, Sabine Riethdorf, Andra Kuske, Gerd Leitinger, Klaus Pantel, Thomas Kroneis, Peter Sedlmayr

**Affiliations:** 1 Institute of Cell Biology, Histology and Embryology, Medical University of Graz, Graz, Austria; 2 Department of Therapeutic Radiology and Oncology, Medical University of Graz, Graz, Austria; 3 Institute of Pathology, Medical University of Graz, Graz, Austria; 4 Fraunhofer Institute for Toxicology and Experimental Medicine ITEM, Regensburg, Germany; 5 Department of Tumor Biology, University Medical Center Hamburg-Eppendorf, Hamburg, Germany; 6 Sahlgrenska Cancer Center, University of Gothenburg, Gothenburg, Sweden

## Abstract

Enumeration and especially molecular characterization of circulating tumour cells (CTCs) holds great promise for cancer management. We tested a modified type of an *in vivo* enrichment device (Catch&Release) for its ability to bind and detach cancer cells for the purpose of single-cell molecular downstream analysis *in vitro*. The evaluation showed that single–cell analysis using array comparative genome hybridization (array-CGH) and next generation sequencing (NGS) is feasible. We found array-CGH to be less noisy when whole genome amplification (WGA) was performed with Ampli1 as compared to GenomePlex (DLRS values 0.65 vs. 1.39). Moreover, Ampli1-processed cells allowed detection of smaller aberrations (median 14.0 vs. 49.9 Mb). Single-cell NGS data obtained from Ampli1-processed samples showed the expected non-synonymous mutations (deletion/SNP) according to bulk DNA. We conclude that clinical application of this refined *in vivo* enrichment device allows CTC enumeration and characterization, thus, representing a promising tool for personalized medicine.

Tumour metastasis is the advanced stage of cancer and accounts for approximately 90% of cancer-related deaths^1^. In the current model of tumour progression circulating tumour cells (CTCs) play an important role as the presence of CTCs has been correlated with metastatic relapse and disease progression^2^,^3^. In addition, the number of CTCs is a strong prognostic factor correlated with outcomes and superior to other variables such as PSA in progressive castration-resistant prostate cancer^4^. CTCs are thought to carry important molecular information from the primary tumour or metastatic sites^5^ so that CTCs harbour the potential value of a biomarker for monitoring genetic cancer progression^6^.

Although the diagnostic impact of CTC analysis may be considerable, their extremely low concentration makes it difficult to exploit their full potential^7^. Various technologies have been used for detection, enumeration, and isolation of CTCs from peripheral blood of patients^8^. So far, however, batch sampling of 10 ml of peripheral blood remains a limitation for many methods leading to suboptimal sensitivity for detection of CTCs^9^. Furthermore, batch sampling requires more or less continuous CTC turnover, which in fact might be neither continuous nor uniform, thus introducing additional bias. Also, CTCs may be quite fragile and escape CTC analyses during multi-step *ex vivo* isolation procedures^10^, this causing a process-related bias. In contrast to batch sample-based enrichment techniques, *in vivo* enrichment of CTCs may evade some of the bias.

The CellCollector CANCER01 (DC01, GILUPI) is a CE-approved medical device that uses antibodies against the epithelial cell adhesion molecule (EpCAM) for isolating CTCs directly from peripheral blood *in vivo*^11^. When compared in the same patient cohort the DC01 resulted in a higher number of patients positive for CTCs than CellSearch^12^ underlining DC01’s potential for CTC analysis. However, DC01 was not designed for molecular genetic characterization beyond CTC enumeration. To enable downstream analysis at the single-cell level we tested a novel device allowing recovery of attached cells [CellCollector CANCER03 (Catch & Release, C&R, GILUPI)] which is not yet CE-certified for clinical application, thus currently allowing for *in vitro* use only.

In the present study, we tested if the C&R which is also based on cell enrichment by EpCAM capture, allows isolation and recovery of single tumour cells *in vitro*. For this purpose, we investigated the charging and detachment performance of the CellCollector C&R *in vitro* using tumour cells suspended at different cell densities either in PBS or peripheral blood. In order to test the compatibility of the recovering protocol with downstream analysis for cell characterisation we amplified single cells recovered from the C&R using two whole genome amplification (WGA) strategies and analysed the amplified single-cell products using comparative genomic hybridization (array-CGH) and next generation sequencing (NGS).

## Results

### Catch and release performance of cells enriched by the CellCollector C&R

**Figure 1:**
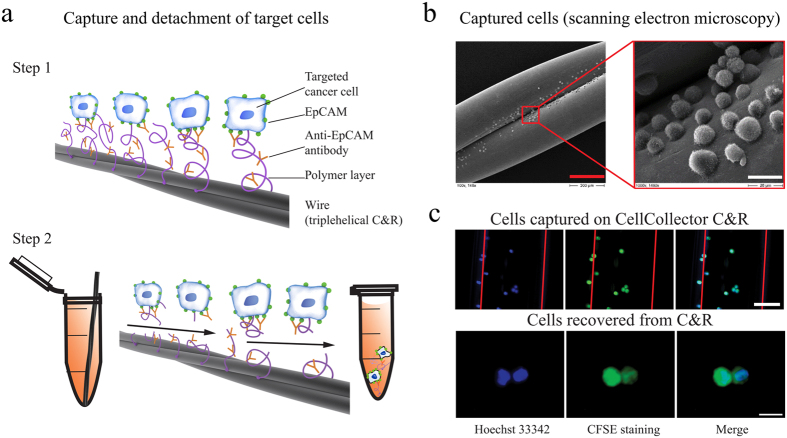
Principle and performance of CellCollector Catch and Release (C&R). (**a**) Schematic composition of the C&R detector. Cells are captured by anti-EpCAM antibodies (step 1) and released by enzymatic disintegration of the polymer layer (step 2) (drawing by Georg Peinhaupt). (**b**) Scanning electron microscopic image of cells captured by the C&R, scale bars 200 μm (red) and 20 μm (white), respectively. (**c**) Captured HT-29 cells stained with Hoechst 33342 (blue) and CFSE (green), red lines indicate the borders of the functionalized wire, scale bar: 50 μm.

**Figure 2:**
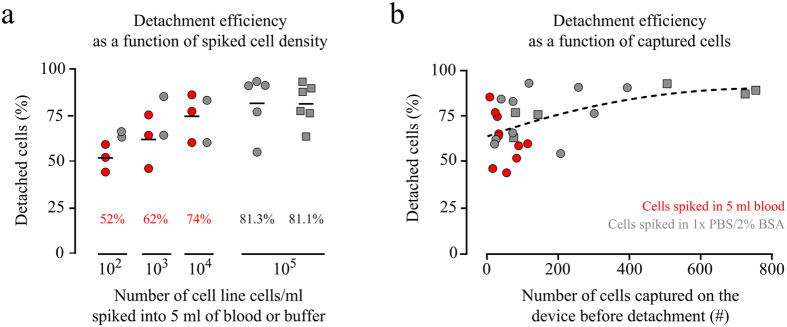
Performance of CellCollector C&R using cultured cancer cells. (**a**) We spiked HT-29 (circles) and LNCaP (squares) cells at densities of 100 to 100,000 cells per ml buffer (grey) or blood (red) and allowed the CellCollector to capture cells during 30 min incubation. Before and after cell detachment the cells on the C&R devices were counted and detachment efficiencies calculated. Detachment efficiencies shown as a function of spiked cell density (**a**) and number of captured cells before detachment (**b**), respectively. Horizontal bars (**a**) indicate mean detachment efficiencies; dashed line is to guide eye only (**b**).

One of the disadvantages of the currently used *in vivo* enrichment devices is that captured cells firmly attach to the wire preventing CTCs to be recovered for further analysis. In contrast, the CellCollector C&R is coated with a polymer layer susceptible to enzymatic treatment (Fig. 1a and Supplementary Table S1). Therefore, captured cells can be detached from the wire and subjected to molecular analysis down to single cell level. When exposed to high target cell concentrations in PBS/2% BSA (i.e. 10^5^ cells/ml) *in vitro*, most cells were found being captured in the groove of the C&R (Fig. 1b). We investigated the C&R’s capacity of isolating and detaching pre-stained cells (Fig. 1c) by spiking 1·10^2^, 1·10^3^, and 1·10^4^ HT-29 cells per ml in 5 ml of peripheral blood (n = 3 each). Thirty minutes of exposure to the wire resulted in 7–113 isolated cells across all applied spiking protocols (for details see Supplementary Table S2). When forwarded to enzymatic treatment re-evaluation of the slides yielded average detachment efficiencies of 52%, 62%, and 74% (Fig. 2a). Control spiking experiments of 1·10^2^, 1·10^3^, and 1·10^4^ HT-29 cells per ml in PBS/2% BSA (n = 2 each) resulted in average detachment rates of 65%, 75%, and 72%, respectively. Exposure of the C&R to 5·10^5^ HT-29 (n = 5) and LNCaP cells (n = 6) in PBS/2% BSA resulted in 117–393 and 74–754 isolated cells, respectively. The detachment of cells performed similar in both cell lines with mean efficiencies of 81.3% (range 54.9%–93.2%) and 81.1% (range 63.51%–92.87), respectively (Fig. 2). Selected recovered cells (Fig. 1c) were forwarded to single-cell isolation by means of micromanipulation.

### Detachment from C&R does not affect single-cell array-CGH quality

**Figure 3:**
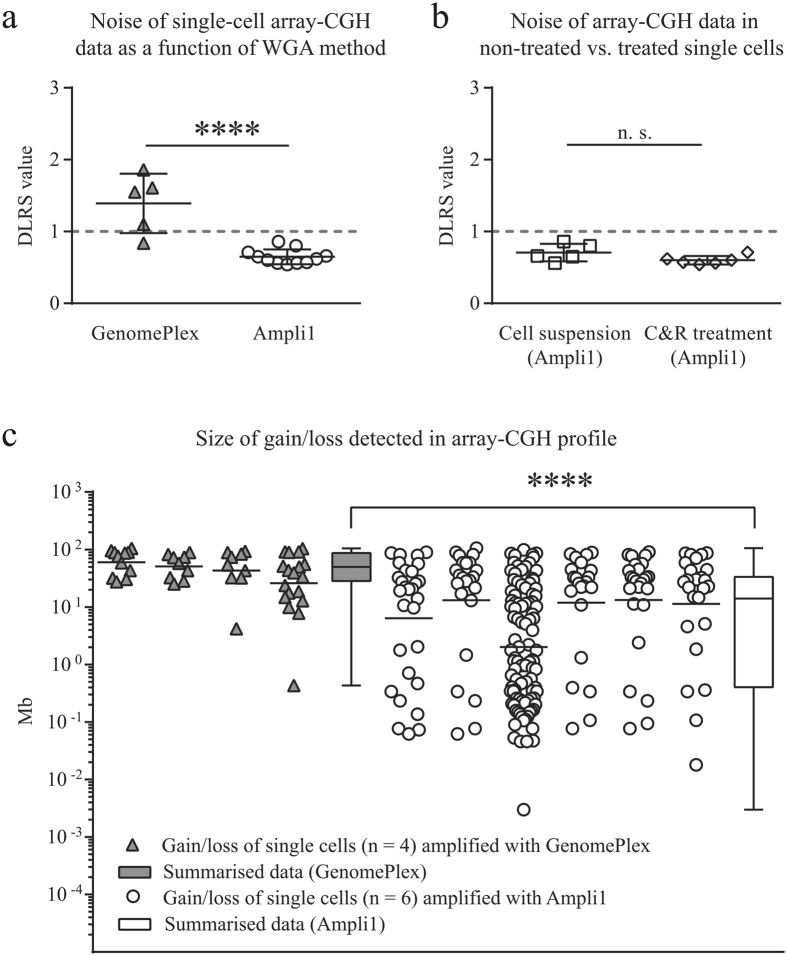
Quality assessment in single-cell analysis based on array-CGH data. Plots of derivative log ratio spread (DLRS) values indicating noisiness in log ratio data of single cells are shown. (**a**) Single cells amplified with GenomePlex yielded higher DLRS values as cells amplified with Ampli1indicating poor signal-to-noise properties associated with less accurate CNV detection (unpaired *t*-test, p < 0.0001). (**b**) Using Ampli1, no difference (p = 0.16) was seen between single cells obtained from cell suspensions and cells that were recovered from C&R. All single cells amplified with Ampli1 have DLRS values < 1 (dashed grey line). (**c**) Single cells amplified by means of Ampli1 (white circles) yielded a higher number of gains and losses at low sizes as compared to WGA4-amplified cells (grey triangles). This was highly significant when we analyzed the aberration detection performance based on the WGA-method (white bar: Ampli1; grey bar: GenomePlex) used (p < 0.0001, Mann-Whitney *U* test).

Next, we amplified single-cell DNA of the micromanipulated cells (tumour cell lines) mentioned above by two different WGA technologies, i.e. Ampli1 and GenomePlex, and assessed their performance on multiple levels. First, we performed QC-PCR^13^ to identify cells suitable for downstream analysis. These data revealed that the number of cells yielding satisfying amplification products was higher when we used Ampli1 instead of GenomePlex (5/8 vs 3/8 single cells, respectively, Supplementary Fig. S1). WGA products with sufficient quality were then subjected to array-CGH. The resulting derivative log_2_ ratio spread (DLRS) values were used to evaluate the performance of the two WGA protocols and sampling procedures at the array level yielding a much more detailed picture. Generally, non-amplified genomic DNA scored lowest DLRS values (~0.3). Compared to that, Ampli1 and GenomePlex amplified single cells showed increased noise levels with Ampli1-processed cells yielding significantly lower DLRS values than cells processed by GenomePlex [mean DLRS 0.65 (n = 11) vs 1.39 (n = 5); unpaired *t*-test, p < 0.0001, Fig. 3a]. In addition, Ampli1-processed cells showed higher concordance with the genomic DNA reference sample (Supplementary Fig. S2). Moreover, we saw no significant difference between single cells directly isolated from cell suspension and those detached from C&R [DLRS mean values 0.71 (n = 5) vs. 0.60 (n = 6), p > 0.05; Fig. 3b], nor was there a cell line-specific difference (Supplementary Fig. S3). To further specify noise-related effects on the array-CGH profiles we had a closer look on HT-29 cells after GenomePlex (n = 4) and Ampli1 (n = 6) amplification. We detected a higher number of aberrations in Ampli1-processed cells (mean 37 vs 12) and significantly shorter aberrations as compared to GenomePlex-processed cells (mean 14.01 vs 49.9 Mb, p < 0.0001; Fig. 2c, Supplementary Tables S3 and S4). In addition, we observed single cell heterogeneity identifying microdeletions present in single cells but not in the bulk sample (Supplementary Figs S4 and S5, Supplementary Tables S3 and S4).

### Detection of non-synonymous mutations of C&R recovered single cells

Next, we forwarded non-amplified genomic DNA (gDNA) and C&R-treated and Ampli1-processed single cells (LNCaP and HT-29 cell line cells, ten and five cells, respectively) to targeted NGS. Sequencing yielded at minimum 1 million reads across all single cells with most abundant reads in the expected range between 130 and 139 bp (87–93% of reads on target; >90% above AQ20) and only a few amplicons to drop out (Supplementary Fig. S6).

For LNCaP cells, sequencing data unveiled a codon 6 frameshift mutation in *PTEN* at 100% mutant allele frequency and the *TP53* P72R polymorphism for all ten single cells. Furthermore, we found a mutation in *SMAD4* in eight of ten cells at mutant allele frequency rates ranging from 19% to 37%. Two single cells showed mutations in additional three genes (*CTNNB1, FBXW7, SMO*) at low mutation frequency (range 13% to 15%).

All five sequenced single HT-29 cell samples yielded homozygous *TP53* (R273H) and *SMAD4* (Q311) mutation at 100% mutant allele frequency. We detected *KIT* (M541L) and *APC* (E1554E) in all as well as *BRAF* (V600E) mutations in four of five single cells with their mutation frequencies being similar to HT-29 bulk DNA. One cell presented with a second *SMAD4* (H530R) mutation albeit at a low allele mutation frequency of 18%. Additionally, we found noncoding SNPs in single cells of both cell lines. Tables 1 and 2 summarise the sequencing data of LNCaP and HT-29 cells, respectively.

## Discussion

Recent clinical data^14^ and our own data (work in EU-FP7-consortium CTC-SCAN, to be reported elsewhere) suggest that *in vivo* enrichment of CTCs using CellCollector DC01 results in detection of higher CTC numbers and increased sensitivity for detection in patients as compared to CellSearch, which is the current gold standard for CTC enumeration. Based on the promising data regarding CTC isolation we investigated if we can successfully link the *in vivo* isolation approach with single-cell downstream analysis. In this study we report our data regarding a new version of an anti-EpCAM-coated detector, called C&R (for catch and release), designed (but not yet clinically certified) for CTC enrichment directly from peripheral blood. It resembles the CellCollector DC01 regarding its CTC capture principle but, in addition, comes with some advantages (summarized in Supplementary Table S1) with its cell detachment option being the most important. The rationale of this study was to examine if this setting would technically allow CTC characterisation beyond enumeration ideally; such a characterisation could be indicative for treatment decision^15^.

First, we tested the efficiency of the C&R to isolate and detach target cells expressing EpCAM. Exposing the device to high cell densities (i.e. 10^5^ cells/ml) resulted in cells covering the device across the whole length (Fig. 1b). Detachment efficiencies were >80% for HT-29 and LNCaP cell line cells indicating, as expected, no difference between cells of different EpCAM expression levels. When exposed to less target cells (500–50 000) spiked into 5 ml of peripheral blood, the isolated number of cells ranged from 7 to 113 cells (see Supplementary Table S2) showing no correlation with spiked numbers of HT-29 cells. Towards lower target cell densities the detachment efficiency decreased to range between >50% and 75% (Fig. 2a).

We were able to perform single-cell DNA analysis after recovering LNCaP and HT-29 cells from the C&R device by enzymatically disintegrating its polymer layer (Fig. 1). Importantly, the procedure of recovering single cells from the C&R for downstream analysis did not affect the DNA quality (Fig. 3b). However, we found DNA quality to be significantly affected by the choice of the WGA method used for single cell amplification. This was evident across analysed cell lines as DLRS values, which reflect noisiness in log ratio data (where increased values correlate with poor signal-to-noise properties) hampering accurate detection of aberrations: Single cells amplified with linker adapter-based Ampli1 showed >2.5-fold reduction of background noise (Fig. 3a, Supplementary Fig. S2). As a consequence of this we achieved a significantly higher resolution in array-CGH profiles when using Ampli1 compared to GenomePlex (Fig. 3c, Supplementary Tables S3 and S4). This probably points at a shortcoming of the heat-fragmentation based GenomePlex procedure not seen in earlier experiments done on low-template analysis (containing several nuclei) of syncytial nuclear aggregates^16^.

Using Ampli1-based WGA in NGS we were able to add another layer of high resolution molecular genetic analysis to the array-CGH screening. We detected a number of sequence variants in the single cells, which were matching the data obtained from non-amplified bulk cell DNA (Tables 1 and 2). Notably, the frame shift mutation in *PTEN* turned out to be homozygous in all our single LNCaP cells (Table 1), whereas it was previously reported to be heterozygous^17^. Some cells showed mutations in additional genes (*CTNNB1, FBXW7, SMO*) at low mutation frequency (range 13 to 15%), which may be partly due to a true mutation event occurring at one allele in the hypertriploid/hypotetraploid cells, polymerase error during WGA or sequencing errors based on erroneous target enrichment^18^.

Growing knowledge in the field of cancer genetics will be constantly used to improve disease monitoring and treatment^19^ with CTC analysis evolving from mere enumeration towards molecular characterization. Promising targets driving cancer progression, drug resistance, and relapse such as androgen receptor variants, loss of *PTEN*, and *ETS* gene rearrangement have already been identified and liquid biopsy might provide a feasible non-invasive and low-risk access to optimized treatment^20^,^21^,^22^.

Our data show that target cells can be efficiently detached from the CellCollector C&R. Moreover, processed single cells yielded high quality DNA suitable for a combined screening-based (array-CGH) and target-specific (NGS) analysis approach. The data from our *in vitro* study provide evidence of C&R’s potential for clinical application, hence, its clinical application contributing to drive cancer treatment towards personalized medicine.

## Methods

### Sample collection and processing

This study was approved by the ethics committee of the Medical University of Graz (25–240 ex 12/13). Peripheral blood for spiking experiments was sampled from healthy controls. All methods were performed in accordance with the relevant guidelines and regulations.

### Cell lines and evaluation of CellCollector C&R

Human cancer cell lines HT-29 (colon, kindly provided by GILUPI), LNCaP (prostate, kindly provided by Martine Mazel, Laboratory of Rare Circulating Human Cells, Montpellier, France), and PC-3 (prostate, obtained from ATCC) were cultured as recommended by the distributors. Cells were pre-stained with carboxyfluorescein succinimidyl ester (CFSE, Life Technologies; 5 μM in DMSO) and Hoechst 33342 and rinsed twice in 1x PBS. Stained cells were resuspended at different concentrations ranging from 100 to 100,000 cells/ml in 5 ml of PBS/2% BSA or peripheral blood. The latter was rinsed and restored to initial blood volume with PBS to avoid clotting of blood group antigen expressing HT-29 cells. The CellCollectors were charged with target cells by incubation with 5 ml spiked cell suspension at room temperature (RT) in a 9 ml reaction tube (Venosafe) on a horizontal roller mixer (SRT6D, Stuart Scientific) at 5 rpm for 30 min. For cell detachment, the C&R was incubated in pre-warmed 1x release buffer (GILUPI) at 37 °C for 10 min followed by agitation at 200 rpm on a plate shaker (DELFIA, Perkin-Elmer) at RT for 15 min. After centrifugation at 300× *g* (Heraeus Megafuge 40R, Thermo Fisher) for 10 min we gently removed the C&R device and centrifuged again. Collected cells were rinsed twice in 1x PBS/2% BSA, resuspended in 50 μl and transferred to chamber slides (Nunc). We counted the cells on the C&R before and after the detachment treatment under florescent microscope (Observer Z1, Zeiss) to evaluate cell detachment efficiency. We micromanipulated recovered cells using an inverted microscope (Axiovert M 200, Zeiss) as previously reported^23^. In short, we transferred 1 μl 1x PBS containing single cells or cell pools into caps of 200 μl PCR tubes for subsequent WGA using Ampli1™ WGA Kit (Silicon Biosystems) or GenomePlex Kit (Sigma). For the latter the PCR caps were preloaded with 9 μl of nuclease-free water. Samples were immediately centrifuged shortly, forwarded to WGA by Ampli1 or stored at −80 °C for later amplification using GenomePlex. Genomic DNA was extracted from cell lines using the DNeasy Blood and Tissue Kit (Qiagen) as described by the manufacturer. DNA concentration and purity were determined using a Nanodrop spectrophotometer (Thermo Fisher).

### Single-cell whole genome amplification

Amplification using GenomePlex was performed as previously described with minor modifications^24^,^25^. Briefly, cells were digested in 1x single cell lysis and fragmentation buffer containing proteinase K (Sigma), followed by GenomePlex library preparation. Amplification was performed by adding 7.5 μl of 10 × amplification master mix, 48.5 μl nuclease-free water, and 5 μl WGA DNA polymerase.

Ampli1 amplification was performed according to the manufacturer’s protocol and published before^26^. Briefly, samples collected in 1 μl of PBS were lysed with 2 μl lysis reaction mix (42 °C, overnight). DNA was digested after adding 2 μl of digestion reaction mix at 37 °C for 3 h. Following enzyme inactivation (65 °C for 5 min), 5 μl of ligation mix containing pre-annealed adapter nucleotides were ligated to the fragmented DNA at 15 °C overnight. Finally, 40 μl of Primary PCR Reaction Mix was added to each sample and amplified as published^26^.

WGA products were purified using GenElute PCR Clean-Up Kit (Sigma) and subjected to a quality control multiplex PCR (QC-PCR) as described previously^13^,^27^. Samples not passing the QC criteria (<3 of 4 PCR products) were excluded from array-CGH and NGS analysis. Ampli1-processed samples were re-amplified for array-CGH and NGS as published recently^28^.

### Array comparative genomic hybridization (array-CGH)

Each purified sample was labelled with the Bioprime Array CGH Genomic Labeling System (Life Technologies) as described previously^24^. Briefly, dCTP-Cy5 labelled sample DNA and dCTP-Cy3 labelled reference DNA (both GE Healthcare) were purified with Amicon Ultracel-30 filters (Millipore) and mixed in equal amounts of 250 ng for hybridization. We ran array-CGH on oligonucleotide-based SurePrint G3 Human CGH 8 × 60 K Microarrays (Agilent Technologies), processed the images with Feature Extraction software and performed data analysis using Agilent Genomic Workbench Lite Edition 7.0.4.0 (Agilent). Aberrations were detected using the ADM-2 algorithms with a threshold of eight and Centralization Algorithm set to a threshold of 6.0 with a bin size of 10. Calls needed to span at least three consecutive probes; the minimum absolute average was set to log2 ratio of 0.25. We used derivative log ratio spread (DLRS) values to assess the quality of array-CGH profiles^29^.

Circos plots showing the aberrations of single cells from LNCaP and PC-3 cell lines were generated using open access circos software (http://www.circos.ca).

### Library preparation and Ion Torrent targeted next generation sequencing (NGS)

We amplified 10 ng DNA in Ion AmpliseqHiFi Master Mix using the multiplexed Ampli1 CHPCustom Beta primer panel covering 2265 COSMIC hot spot regions across 315 amplicons of 50 cancer-related genes (kindly provided by Silicon Biosystems; Supplementary Table S5). Quality and concentration of the amplicon pools were determined using the High Sensitivity DNA Kit on a 2100 Bioanalyzer (both Agilent). The amplicons were ligated to adapters containing Ion Express Barcode Adaptors Kit (Life Technologies) according to the manufacturer’s instructions. Prepared libraries were quantified by qPCR using the Ion Library Quantitation Kit (Life Technologies). Clonal amplification of the barcoded DNA libraries was carried out using Ion OneTouch 200 kit and libraries were sequenced on an Ion Proton sequencer using the Ion P1 chip (PGM sequencer; Ion Torrent).

All samples were sequenced to a depth of >1 million reads, with reads in the expected range between 90 and 130 bp, 87–93% of reads on target and more than 90% above AQ20. Mapping to hg19 and variant calling was performed using the open source Ion Torrent Suite Software (Life Technologies). All called variants were annotated using open source software (ANNOVAR^30^; SnpEff 31) and custom Perl scripts. All coding, non-synonymous mutations were further evaluated and visually inspected in Integrated Genome Viewer^32^ and variant calls resulting from technical read errors or sequence effects were excluded from the analysis. The threshold of detected variant frequency was set to 10% mutated alleles among all samples.

### Scanning electron microscopy (SEM) images of cells captured by CellCollector C&R

A CellCollector C&R loaded with HT-29 cells was subjected to SEM sample processing as previously described^33^,^34^. Briefly, the devices were rinsed in 1x PBS, fixed in 0.1 M sodium cacodylate buffer (pH 7.4) containing 2% formaldehyde and 2.5% glutaraldehyde, and rinsed 1x in PBS. The cells were post-fixed in the 0.1 M sodium cacodylate buffer containing 2% osmium tetroxide, rinsed again, and dehydrated in an ascending ethanol series followed by acetone. The samples were dried using a BalTec CPD 030 critical point dryer, sputter coated using a Leica SCD 500 sputter coater, and visualized using a Zeiss DSM 950 scanning electron microscope.

### Statistical methods

Unpaired two-tailed *t* test (DLRS) and Mann-Whitney *U* test (aberration size) were performed using GraphPad Prism 6.0.

## Additional Information

**How to cite this article**: Chen, S. *et al*. Catch and Release: rare cell analysis from a functionalised medical wire. *Sci. Rep.* **7**, 43424; doi: 10.1038/srep43424 (2017).

**Publisher's note:** Springer Nature remains neutral with regard to jurisdictional claims in published maps and institutional affiliations.

## Supplementary Material

Supplementary Information

## Figures and Tables

**Table 1:** Non-synonymus mutation frequencies of Ampli1-amplified single LNCaP cells after recovery from the C&R detector as well as non-amplified genomic DNA of LNCaP cell line cells.

Gene	Mutation	COSMIC ID	Mutation frequency (%)											
			Single LNCaP cells											gDNA^a^
			#1	#2	#3	#4	#5	#6	#7	#8	#9	#10	**mean**	
*PTEN*	del^b^	—	100	100	100	100	100	100	100	100	100	100	**100**	100
*TP53*	P72R	COSM250061	92	94	93	93	92	94	91	94	93	94	**93**	95
*SMAD4*	L533P	COSM189744	23	30	24	20	22	37	19	22	0	0	**20**	22
*CTNNB1*	M12V	—	0	0	0	0	0	0	0	0	13	0	**1**	0
*FBXW7*	A503T	—	0	0	0	13	0	0	0	0	0	0	**1**	0
*SMO*	A401V	—	0	0	0	15	0	0	0	0	0	0	**2**	0

^a^Genomic DNA, non-amplified.

^b^Deletion.

**Table 2:** Non-synonymus mutation frequencies of Ampli1-amplified single HT-29 cells after recovery from the C&R detector as well as non-amplified genomic DNA of HT-29 cell line cells.

Gene	Mutation	COSMIC ID	Mutation frequency (%)						
			Single HT-29 cells						gDNA^a^
			#1	#2	#3	#4	#5	mean	
*KIT*	M541L	COSM28026	24	27	23	24	30	**26**	26
*APC*	E1554E^a^	COSM33818	84	53	57	49	61	**61**	65
*BRAF*	V600E	COSM476	23	34	25	0	25	**21**	28
*TP53*	R273H	COSM10660	100	100	100	100	100	**100**	100
*SMAD4*	Q311^a^	COSM14163	100	100	100	100	100	**100**	100
*SMAD4*	H530R	—	18	0	0	0	0	**2**	0

^a^Mutated to stop codon.
