# Supplementary material for: Catch and Release: rare cell analysis from a functionalised medical wire
Source: Sci Rep. 2017 Feb 24;7:43424. doi: 10.1038/srep43424 (PMC5324062; doi:10.1038/srep43424)
Supplement: Supplementary Information [file srep43424-s1.pdf]

Catch and Release: rare cell analysis from a functionalised medical wire

Shukun Chen[1], Amin El-Heliebi[1], Gerlinde Tauber[2], Tanja Langsenlehner[2], Michaela Pötscher[2], Karl Kashofer[3], Zbigniew T. Czyż[4], Bernhard Polzer[4], Sabine Riethdorf[5], Andra Kuske[5], Gerd Leitinger[1], Klaus Pantel[5], Thomas Kroneis[1,6*], Peter Sedlmayr[1]

1. Institute of Cell Biology, Histology and Embryology, Medical University of Graz, Graz, Austria
2. Department of Therapeutic Radiology and Oncology, Medical University of Graz, Graz, Austria
3. Institute of Pathology, Medical University of Graz, Graz, Austria
4. Fraunhofer Institute for Toxicology and Experimental Medicine ITEM, Regensburg, Germany
5. Department of Tumor Biology, University Medical Center Hamburg-Eppendorf, Hamburg, Germany
6. Sahlgrenska Cancer Center, University of Gothenburg, Gothenburg, Sweden

*corresponding author: Thomas Kroneis; Harrachgasse 21, 8010 Graz, Austria; tel: +43 (0)316 380 4236; fax: +43 (0)316 380 9625; thomas.kroneis@medunigraz.at

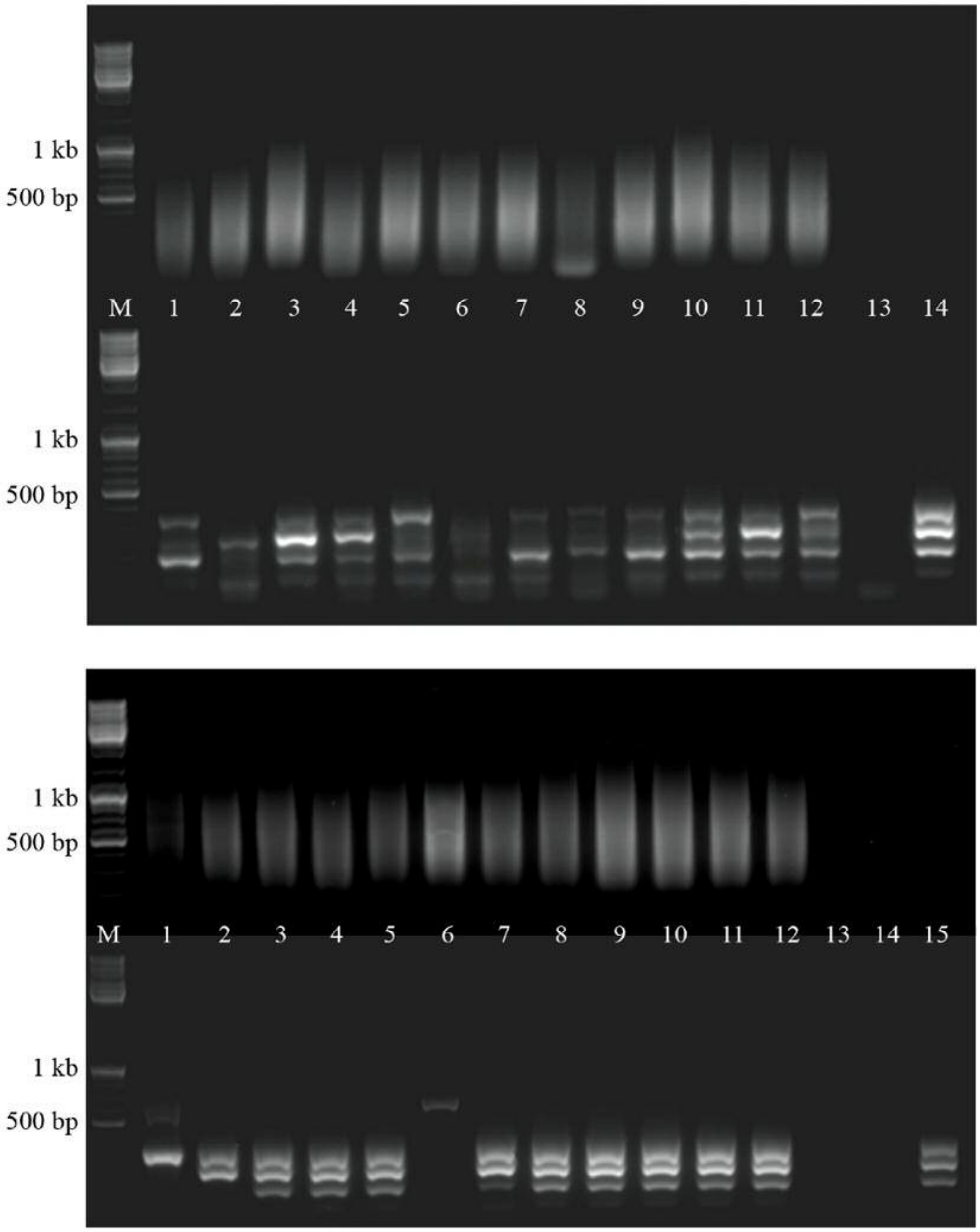

Supplementary Figure S1

WGA smears and quality control PCR of single cells amplified by GenomePlex or Ampli1. To assess the performance of the WGA methods used, we performed 4plex PCR on the amplified single-cell samples. PCR products of this QC-PCR and aliquots of their corresponding WGA templates were run on an agarose gel. WGA smears (range 300 - > 1000 bp; maximum at about 300-500 bp) and the number of positive QC-PCR bands (≥ 3) indicated WGA products of good quality. *Upper panel:* #1-8: single cells; #9-12: cell pools (n = 5); #13: negative control; #14: positive control. Samples of #3-5 and #10-12 showed 3 or 4 bands. *Lower panel:* #1-8: single cells; #9-12: cell pools (n = 5); #13 &14: negative control; #15: positive control. Sample of #3-5 and #7-12 showed 3 or 4 bands; bp: base pairs.

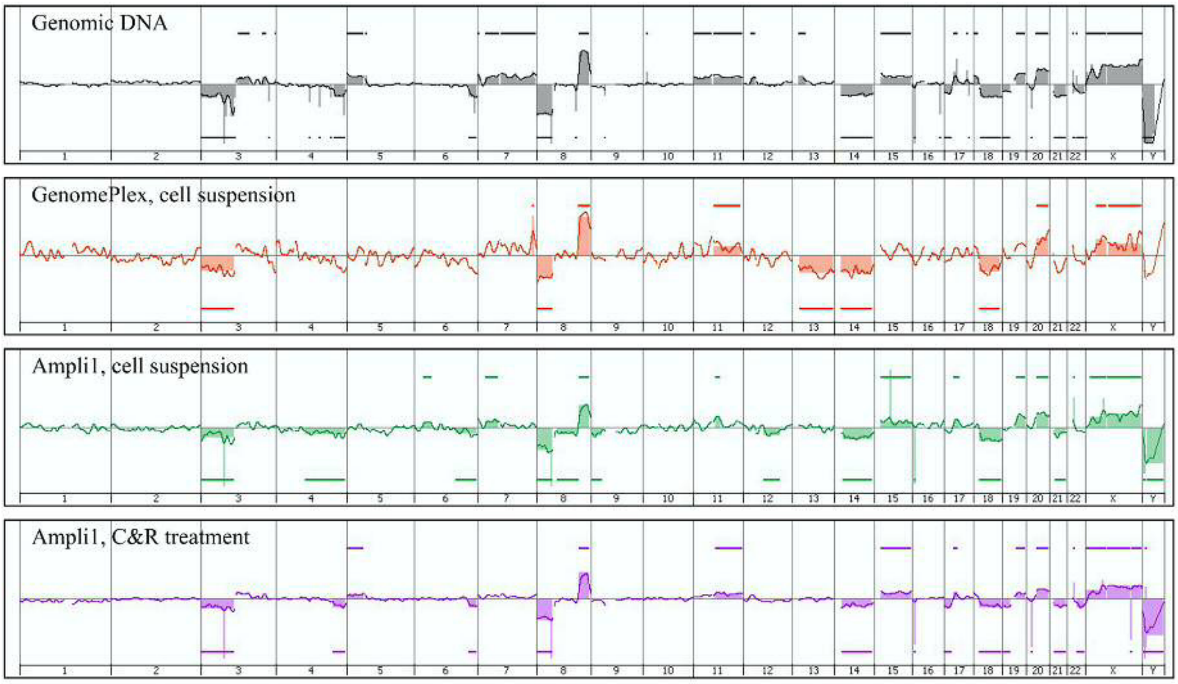

Supplementary Figure S2 Genome-wide array-CGH profiles of HT-29 cells generated using unamplified gDNA and single cells. Gains and losses of non-amplified genomic DNA (black), single cell from cell-suspension amplified by GenomePlex (orange), single cell from cell suspension amplified by Ampli1 (green) and single cells recovered from C&R detector amplified by Ampli1 (purple). All samples were co-hybridised with female reference DNA.

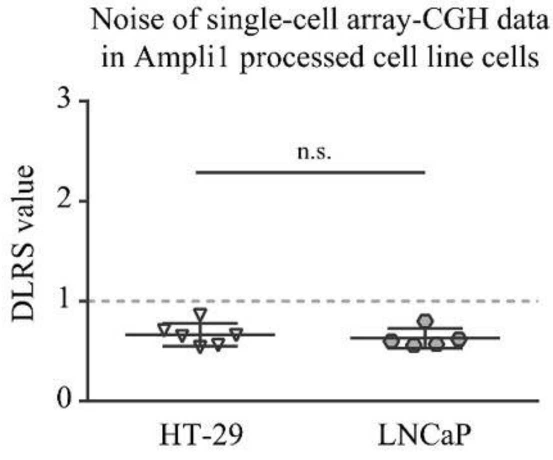

Supplementary Figure S3 Quality assessment of single cell array-CGH of different cell lines cells post Ampli1 amplification. Plots of derivative log ratio spread (DLRS) values indicating noisiness in log ratio data of single cells are shown. Using Ampli1, no cell line-specific difference (unpaired *t*-test, $p = 0.62$) was seen at the single-cell level (HT-29 vs. LNCaP). All single cells amplified with Ampli1 have DLRS values < 1 (dashed grey line) indicating these samples to meet the criteria for single-cell array-CGH.

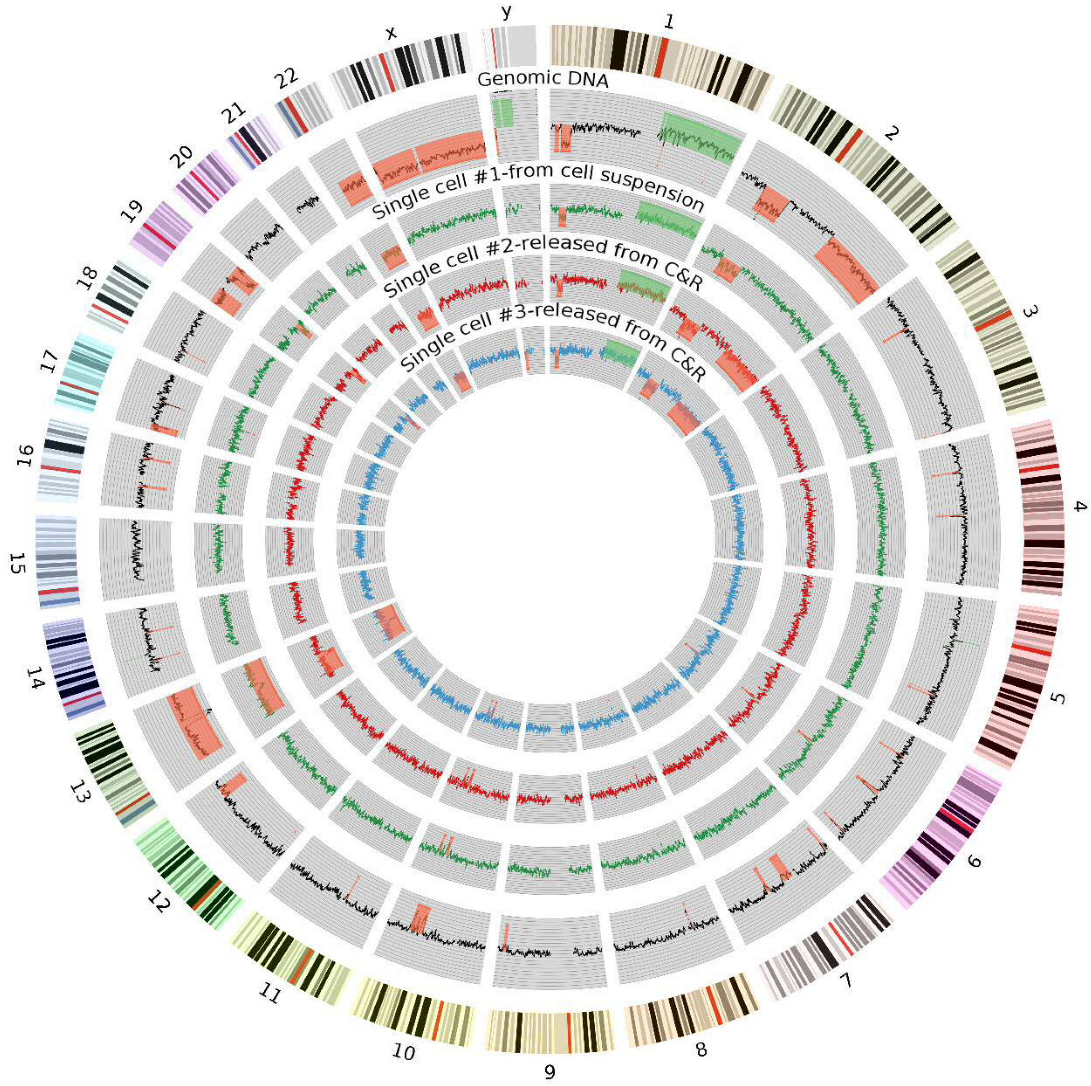

Supplementary Figure S4 Characterization of genome-wide profile of copy number variations in LNCaP prostate cell line (single-cell WGA: Ampli1). Circos plot depicting copy number variations in the genome of LNCaP cells. C&R recovered single LNCaP cells were amplified by Ampli1. The outermost ring is the human chromosome ideogram which is oriented pter-qter in a clockwise direction from chromosome 1 to chromosome X (centromeres are shown in red). Other tracks (from outermost to innermost track) represent: non-amplified genomic DNA (black), a single cell sampled from cell suspension by micromanipulation (green), and two single cells (red, blue) recovered from. Centered splines represent a balanced profile; data points above and below represent gains and losses, respectively. Regions of major copy number changes are highlighted in green (gain) or orange (loss). Nota bene: We used female reference DNA for co-hybridisation with the genomic (male) DNA resulting in X-chromosomal loss and a gain in the y-chromosome. For the single-cell hybridisation we used reference DNA obtained from ten male leukocytes that went through two rounds of amplification resulting in balanced sex chromosome profiles.

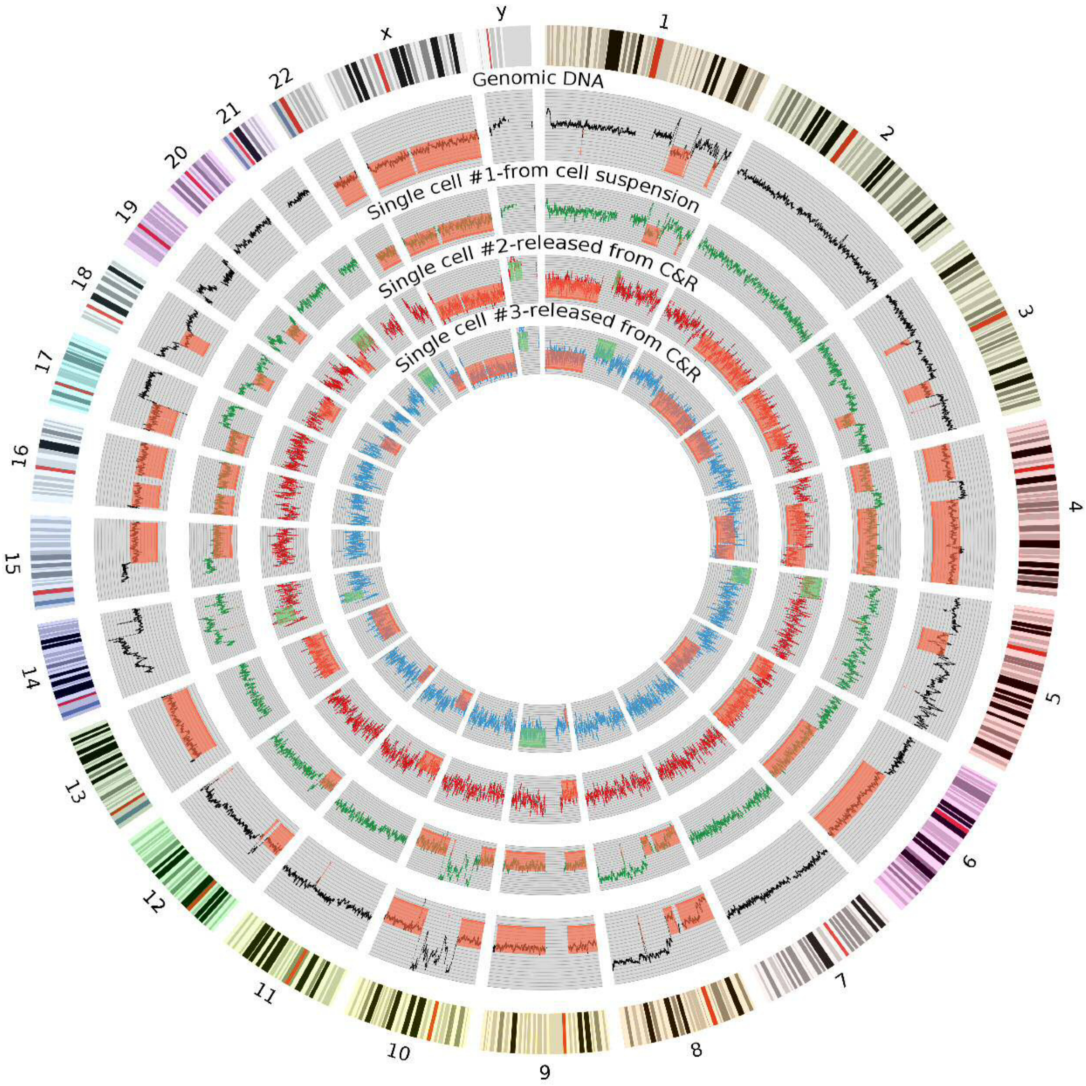

Supplementary Figure S5 Characterization of genome-wide profile of copy number variations in PC-3 prostate cell line. Circos plot depicting copy number variations in the genome of PC-3 cells. C&R recovered single PC-3 cells were amplified by GenomePlex. The outermost ring is the human chromosome ideogram which is oriented pter-qter in a clockwise direction from chromosome 1 to chromosome X (centromeres are shown in red). Other tracks (from outermost to innermost track) represent: non-amplified genomic DNA (black), a single cell sampled from cell suspension by micromanipulation (green), and two single cells (red, blue) recovered from. All samples were co-hybridised with female reference DNA. Centered splines represent a balanced profile; data points above and below represent gains and losses, respectively. Regions of major copy number changes are highlighted in green (gain) or orange (loss).

a

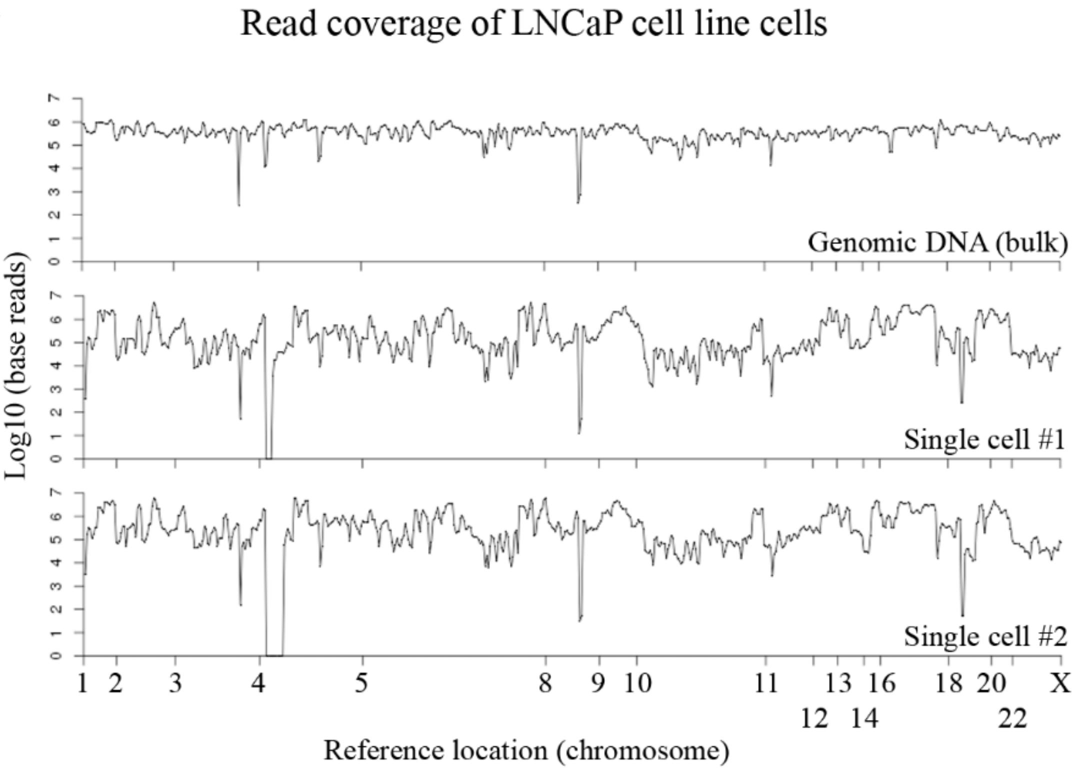

b

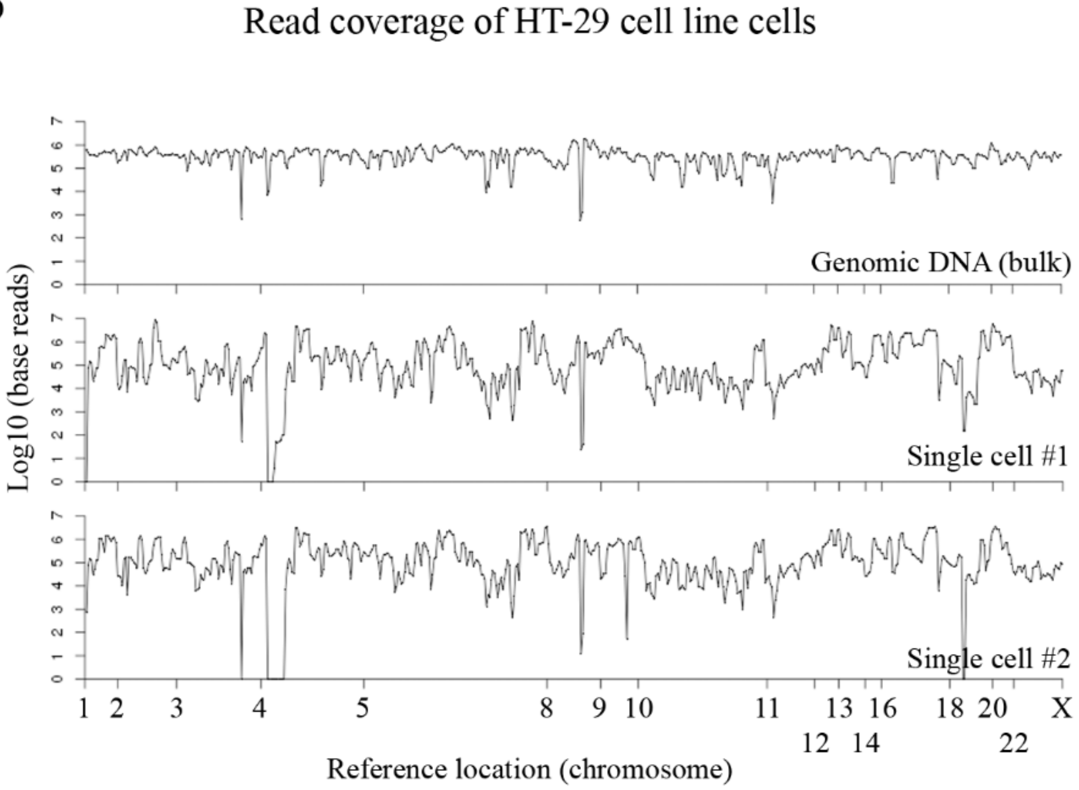

Supplementary Figure S6 Quality control assessments of recovered single cells for NGS analysis. Read coverage overview of genomic DNA and two recovered single cells obtained from LNCaP (top) and HT-29 (bottom) cell line, respectively.

Supplementary Table S1. Comparison of features between CellCollector DC01 and C&R

**Type**	**Functional part**			**CTC release**	**Clinical application**
	Length	Antibody-conjugation material	Structure		
DC01	2 cm	Hydrogel	Single wire	No	Yes (CE certified)
C&R	4 cm	Polymer	Triple wire (helical arrangement)	Yes	No (in vitro use only)

Supplementary Table S2. Cells captured and detached from CellCollector C&R

C&R (#)	Cell line	Spiked cells (#/ml)	Spiking background	Cells on C&R (#) (before detachment)	Cells on C&R (#) (after detachment)	Detached cells (#)	Detachment efficiency (%)
1	HT-29	100	blood	88	36	52	59
2	HT-29	100	blood	54	30	24	44
3	HT-29	100	blood	82	39	43	52
4	HT-29	100	buffer	24	9	15	63
5	HT-29	100	buffer	32	11	21	66
6	HT-29	1,000	blood	31	11	20	65
7	HT-29	1,000	blood	15	8	7	47
8	HT-29	1,000	blood	28	7	21	75
9	HT-29	1,000	buffer	39	6	33	85
10	HT-29	1,000	buffer	71	24	47	66
11	HT-29	10,000	blood	113	45	68	60
12	HT-29	10,000	blood	22	5	17	77
13	HT-29	10,000	blood	7	1	6	86
14	HT-29	10,000	buffer	20	8	12	60
15	HT-29	10,000	buffer	72	12	60	83
16	HT-29	100,000	buffer	117	8	109	93.2
17	HT-29	100,000	buffer	206	93	113	54.9
18	HT-29	100,000	buffer	300	70	230	76.7
19	HT-29	100,000	buffer	256	23	233	91.0
20	HT-29	100,000	buffer	393	36	357	90.8

21	LNCaP	100,000	buffer	74	27	47	64
22	LNCaP	100,000	buffer	142	34	108	76.1
23	LNCaP	100,000	buffer	505	36	469	92.9
24	LNCaP	100,000	buffer	724	91	633	87.4
25	LNCaP	100,000	buffer	754	80	674	89.4
26	LNCaP	100,000	buffer	79	18	61	77

Supplementary Tab. S3. Details of gains and losses (Mb) detected in single HT-29 cells (array-CGH) – sorted according to location within genome

**Chromosome #**	**Aberration**							**Sample**	**Amplification**
	Cytoband	Start	Stop	# of probes	Amp	Del	size (Mb)	(# of cell/genomic DNA)	**method**
chr01	p36.33 - p34.1	1179223	45580317	1046	0.000	-0.292	44.4	7	Ampli1
chr01	p36.12 - p13.2	22837102	114149968	1887	0.497	0.000	91.3	4	GenomePlex
chr01	p12	118214256	118303912	6	2.588	0.000	0.1	7	Ampli1
chr01	q21.1 - q44	143639135	247179291	2247	0.748	0.000	103.5	4	GenomePlex
chr01	q21.1 - q23.2	143720878	158725456	444	0.000	-0.354	15.0	10	Ampli1
chr01	q21.2 - q21.3	148125527	153211494	194	0.000	-0.518	5.1	7	Ampli1
chr01	q21.3	153214463	153217477	3	3.378	0.000	0.0	7	Ampli1
chr01	q31.1	185882529	186223322	4	2.962	0.000	0.3	7	Ampli1
chr01	q42.12	223669592	224099906	10	5.344	0.000	0.4	4	GenomePlex
chr02	p22.3	35213098	35361530	5	2.724	0.000	0.1	7	Ampli1
chr02	p16.3 - p15	48768816	61576198	206	0.547	0.000	12.8	7	Ampli1
chr02	p16.3	50247461	50746410	10	2.691	0.000	0.5	7	Ampli1
chr02	p12	78199429	78768383	8	2.508	0.000	0.6	7	Ampli1
chr02	q21.2	133925196	134018942	3	0.000	-4.621	0.1	9	Ampli1
chr02	q21.3 - q35	136198965	215281055	1392	0.427	0.000	79.1	7	Ampli1
chr02	q31.1	175450840	175711131	7	3.087	0.000	0.3	7	Ampli1
chr02	q36.3 - q37.1	227041180	230975907	77	0.657	0.000	3.9	7	Ampli1
chr03	p26.3 - p11.1	187711	89612764	1653	0.000	-1.210	89.4	1	GenomePlex
chr03	p26.3 - p11.1	187711	89920060	1656	0.000	-0.433	89.7	6	Ampli1
chr03	p26.3 - p11.1	245649	89473737	1649	0.000	-0.383	89.2	5	Ampli1
chr03	p26.3 - p11.1	345339	90336752	1657	0.000	-0.407	90.0	9	Ampli1
chr03	p26.3 - p11.1	1276800	90336752	1644	0.000	-0.727	89.1	3	GenomePlex
chr03	p26.3 - p11.1	1312273	89768983	1638	0.000	-0.453	88.5	8	Ampli1
chr03	p26.3 - p24.3	1312273	17275073	306	0.000	-0.450	16.0	7	Ampli1
chr03	p26.3 - p11.1	1415081	90102363	1638	0.000	-0.627	88.7	gDNA	n.a.
chr03	p26.3 - p11.1	1651376	90336752	1637	0.000	-0.733	88.7	2	GenomePlex
chr03	p26.3 - p11.1	2937570	89768983	1610	0.000	-0.467	86.8	10	Ampli1
chr03	p24.2	24564676	25272714	10	1.825	0.000	0.7	5	Ampli1
chr03	p24.2	24721899	25272714	9	2.433	0.000	0.6	7	Ampli1

Chromosome #	Aberration							Sample	Amplification method
	Cytoband	Start	Stop	# of probes	Amp	Del	size (Mb)	(# of cell/genomic DNA)	
chr03	p24.2	24980387	25135367	3	0.000	-0.835	0.2	7	Ampli1
chr03	p24.1	27826052	28227816	3	3.345	0.000	0.4	7	Ampli1
chr03	p22.2 - p11.1	37384650	89473737	1029	0.000	-0.519	52.1	7	Ampli1
chr03	p21.32 - p14.2	44440141	58626285	437	0.000	-0.458	14.2	gDNA	n.a.
chr03	p14.2	59866479	60703624	21	0.000	-2.321	0.8	7	Ampli1
chr03	p14.2	59901193	60215556	10	0.000	-1.605	0.3	gDNA	n.a.
chr03	p14.2	60267262	60604904	8	0.000	-5.938	0.3	gDNA	n.a.
chr03	p14.2	60267262	60604904	8	0.000	-4.112	0.3	5	Ampli1
chr03	p14.2	60267262	60604904	8	0.000	-4.849	0.3	6	Ampli1
chr03	p14.2	60267262	60604904	8	0.000	-4.148	0.3	8	Ampli1
chr03	p14.2	60267262	60604904	8	0.000	-4.461	0.3	9	Ampli1
chr03	p14.2	60267262	60604904	8	0.000	-3.777	0.3	10	Ampli1
chr03	p14.2	60659263	60916944	7	0.000	-1.691	0.3	gDNA	n.a.
chr03	p14.1	65438127	65632696	5	0.000	-1.584	0.2	gDNA	n.a.
chr03	p12.3 - p11.1	79550595	89473737	108	0.000	-1.546	9.9	gDNA	n.a.
chr03	p12.2	82622792	83271792	7	0.000	-0.553	0.6	gDNA	n.a.
chr03	q11.2 - q26.31	95263583	176491303	1449	0.256	0.000	81.2	10	Ampli1
chr03	q11.2 - q29	95613257	193919798	1766	0.367	0.000	98.3	7	Ampli1
chr03	q11.2 - q21.3	99151488	130338873	590	0.379	0.000	31.2	9	Ampli1
chr03	q11.2 - q22.1	99651232	132096886	616	0.349	0.000	32.4	gDNA	n.a.
chr03	q12.2	102175540	102410841	3	3.787	0.000	0.2	7	Ampli1
chr03	q24 - q25.2	147266685	154715342	134	1.112	0.000	7.4	7	Ampli1
chr03	q26.1 - q26.31	164616150	176491303	203	0.390	0.000	11.9	gDNA	n.a.
chr03	q26.33	181148809	181382033	6	0.000	-0.839	0.2	gDNA	n.a.
chr03	q29	197118367	199288217	60	0.284	0.000	2.2	gDNA	n.a.
chr04	p14	35612319	37803119	37	0.968	0.000	2.2	7	Ampli1
chr04	q13.2	68288073	68543744	8	2.591	0.000	0.3	7	Ampli1
chr04	q21.21 - q35.1	81124287	186681608	1688	0.000	-0.314	105.6	6	Ampli1
chr04	q22.1	91471385	91979910	15	0.000	-0.874	0.5	gDNA	n.a.
chr04	q26	118827701	119194329	4	0.000	-1.116	0.4	gDNA	n.a.
chr04	q31.1 - q31.21	141396594	142269348	20	1.616	0.000	0.9	7	Ampli1

**Chromosome #**	**Aberration**							**Sample**	**Amplification method**
	Cytoband	Start	Stop	# of probes	Amp	Del	size (Mb)	(# of cell/genomic DNA)	
chr04	q31.22 - q31.23	147264214	150112334	59	0.000	-0.630	2.8	gDNA	n.a.
chr04	q31.3	151576229	151723192	4	3.708	0.000	0.1	7	Ampli1
chr04	q31.3 - q32.1	154725220	155378202	12	2.431	0.000	0.7	7	Ampli1
chr04	q32.1 - q35.2	155881609	187505073	498	0.000	-0.398	31.6	8	Ampli1
chr04	q32.1 - q35.2	156851091	189249479	510	0.000	-0.464	32.4	9	Ampli1
chr04	q32.1 - q35.2	157060447	187691464	478	0.000	-0.592	30.6	gDNA	n.a.
chr04	q32.1 - q35.2	157480785	187505073	472	0.000	-0.331	30.0	10	Ampli1
chr04	q32.3	170010519	170056227	3	3.303	0.000	0.0	7	Ampli1
chr04	q34.1 - q35.1	174696391	184073766	117	0.000	-0.602	9.4	7	Ampli1
chr04	q34.1 - q35.1	174766913	184423869	121	0.000	-0.582	9.7	5	Ampli1
chr04	q35.1	183484605	183760601	7	0.000	-1.506	0.3	gDNA	n.a.
chr04	q35.1	184737019	184843006	4	3.887	0.000	0.1	7	Ampli1
chr05	p15.33 - p12	204737	45376406	641	0.407	0.000	45.2	gDNA	n.a.
chr05	p15.33 - p11	1227543	46136124	632	0.272	0.000	44.9	8	Ampli1
chr05	p15.33 - p11	1597400	46136124	625	0.354	0.000	44.5	10	Ampli1
chr05	p15.33 - p13.1	2265049	40668360	522	0.473	0.000	38.4	7	Ampli1
chr05	p15.33 - p12	4216847	45681293	586	0.274	0.000	41.5	5	Ampli1
chr05	p13.2	36644850	36691596	3	3.708	0.000	0.0	7	Ampli1
chr05	q11.1 - q11.2	49725929	53506747	52	0.452	0.000	3.8	gDNA	n.a.
chr05	q21.1	99300776	100401951	17	1.799	0.000	1.1	7	Ampli1
chr05	q23.2 - q23.3	126918464	127878707	19	1.566	0.000	1.0	7	Ampli1
chr05	q31.2 - q35.3	138080491	177567235	773	0.620	0.000	39.5	4	GenomePlex
chr06	p22.3	20542602	22293983	33	1.432	0.000	1.8	7	Ampli1
chr06	p22.1 - p12.3	26232334	47821490	663	0.311	0.000	21.6	6	Ampli1
chr06	p22.1 - p12.3	26232334	47278590	652	0.295	0.000	21.0	9	Ampli1
chr06	p21.33 - p21.2	30146240	37957830	298	0.796	0.000	7.8	4	GenomePlex
chr06	p21.2	38653366	39047545	9	2.390	0.000	0.4	7	Ampli1
chr06	p12.3 - p11.2	46220052	57620687	200	0.714	0.000	11.4	7	Ampli1
chr06	q11.1 - q21	62448434	105555714	622	0.593	0.000	43.1	7	Ampli1
chr06	q12 - q27	65760879	170700061	1737	0.000	-0.657	104.9	1	GenomePlex
chr06	q12 - q13	66761677	73135665	94	1.252	0.000	6.4	7	Ampli1

**Chromosome #**	**Aberration**							**Sample**	**Amplification method**
	Cytoband	Start	Stop	# of probes	Amp	Del	size (Mb)	(# of cell/genomic DNA)	
chr06	q12 - q14.1	66761677	80772362	214	0.426	0.000	14.0	5	Ampli1
chr06	q21 - q27	113248229	170732033	1001	0.000	-0.280	57.5	6	Ampli1
chr06	q23.3 - q24.1	138942686	139107955	3	4.495	0.000	0.2	7	Ampli1
chr06	q24.3 - q27	147702163	169970658	396	0.000	-0.386	22.3	9	Ampli1
chr06	q24.3 - q27	147747964	170732033	407	0.000	-0.576	23.0	gDNA	n.a.
chr06	q24.3 - q27	147747964	170732033	407	0.000	-0.405	23.0	8	Ampli1
chr06	q24.3 - q27	147867199	170631711	402	0.000	-0.419	22.8	10	Ampli1
chr06	q24.3 - q27	148262568	167463414	345	0.000	-0.414	19.2	5	Ampli1
chr06	q25.2 - q26	153387113	163699406	183	0.000	-0.592	10.3	7	Ampli1
chr06	q26	161798317	162090585	6	0.000	-1.421	0.3	gDNA	n.a.
chr06	q27	164493893	165613516	8	2.142	0.000	1.1	7	Ampli1
chr07	p22.3 - p21.3	187615	7365933	132	0.542	0.000	7.2	gDNA	n.a.
chr07	p22.3 - p11.1	731447	57498383	1005	0.357	0.000	56.8	5	Ampli1
chr07	p22.2	3455014	3795836	8	2.381	0.000	0.3	7	Ampli1
chr07	p22.1 - p11.1	7010835	57498383	886	0.538	0.000	50.5	7	Ampli1
chr07	p21.3	11720901	11844417	4	4.075	0.000	0.1	7	Ampli1
chr07	p15.3 - p11.2	20960167	56534393	663	0.372	0.000	35.6	6	Ampli1
chr07	p15.3 - p11.1	21423817	57498383	664	0.383	0.000	36.1	gDNA	n.a.
chr07	p15.3 - p15.2	23537059	26215681	45	1.491	0.000	2.7	7	Ampli1
chr07	p15.2 - p11.2	26371173	56125662	555	0.297	0.000	29.8	9	Ampli1
chr07	p15.1	28070134	28270994	5	3.095	0.000	0.2	7	Ampli1
chr07	q11.21 - q36.3	62153588	158781397	2065	0.393	0.000	96.6	gDNA	n.a.
chr07	q11.21 - q21.11	62471018	77484115	242	0.537	0.000	15.0	gDNA	n.a.
chr07	q11.21 - q36.3	62471018	158291970	2054	0.895	0.000	95.8	1	GenomePlex
chr07	q11.23 - q36.1	76038093	148961160	1631	0.481	0.000	72.9	7	Ampli1
chr07	q11.23 - q21.11	77260956	77728565	12	1.845	0.000	0.5	5	Ampli1
chr07	q11.23 - q21.11	77260956	77728565	12	2.949	0.000	0.5	7	Ampli1
chr07	q21.12	86663788	87010243	9	2.895	0.000	0.3	7	Ampli1
chr07	q22.1	99500188	102343182	99	0.597	0.000	2.8	gDNA	n.a.
chr07	q22.3 - q31.1	106624587	107760886	30	1.678	0.000	1.1	7	Ampli1
chr07	q31.1 - q31.33	108944653	126890763	411	0.252	0.000	17.9	gDNA	n.a.

Chromosome #	Aberration							Sample	Amplification method
	Cytoband	Start	Stop	# of probes	Amp	Del	size (Mb)	(# of cell/genomic DNA)	
chr07	q31.1	111593091	111638854	3	4.621	0.000	0.0	7	Ampli1
chr07	q31.2 - q31.33	116657223	126811276	232	0.960	0.000	10.2	7	Ampli1
chr07	q35	144211503	144369910	4	3.353	0.000	0.2	7	Ampli1
chr07	q35 - q36.1	146519789	150695992	110	2.087	0.000	4.2	3	GenomePlex
chr07	q35 - q36.2	147314780	152396086	128	0.696	0.000	5.1	gDNA	n.a.
chr08	p23.3 - p11.1	211611	43452795	783	0.000	-1.435	43.2	gDNA	n.a.
chr08	p23.3 - p11.1	211611	43291296	781	0.000	-1.873	43.1	1	GenomePlex
chr08	p23.3 - p11.1	211611	43452795	783	0.000	-1.106	43.2	3	GenomePlex
chr08	p23.3 - p11.1	211611	43229710	780	0.000	-1.105	43.0	6	Ampli1
chr08	p23.3 - p11.1	211611	43452795	783	0.000	-1.202	43.2	7	Ampli1
chr08	p23.3 - p11.1	211611	43452795	783	0.000	-1.070	43.2	8	Ampli1
chr08	p23.3 - p11.1	211611	43452795	783	0.000	-1.100	43.2	10	Ampli1
chr08	p23.3 - p11.1	283177	43452795	782	0.000	-1.324	43.2	2	GenomePlex
chr08	p23.3 - p11.1	283177	43452795	782	0.000	-1.065	43.2	5	Ampli1
chr08	p23.3 - p11.1	760001	43229710	773	0.000	-0.997	42.5	9	Ampli1
chr08	p23.1	8721996	8929171	4	3.046	0.000	0.2	7	Ampli1
chr08	p11.23 - p11.22	39378051	39505315	4	0.000	-5.389	0.1	gDNA	n.a.
chr08	p11.23 - p11.22	39428746	39505315	3	0.000	-5.249	0.1	5	Ampli1
chr08	p11.23 - p11.22	39428746	39505315	3	0.000	-6.483	0.1	6	Ampli1
chr08	p11.23 - p11.22	39428746	39505315	3	0.000	-5.269	0.1	7	Ampli1
chr08	p11.23 - p11.22	39428746	39505315	3	0.000	-5.314	0.1	8	Ampli1
chr08	p11.23 - p11.22	39428746	39505315	3	0.000	-5.847	0.1	9	Ampli1
chr08	q11.23 - q24.3	54642612	146031777	1525	0.604	0.000	91.4	4	GenomePlex
chr08	q12.1 - q23.3	55835709	114702528	971	0.000	-0.300	58.9	6	Ampli1
chr08	q21.11	77282375	77538921	3	5.562	0.000	0.3	7	Ampli1
chr08	q22.3 - q23.1	105505760	107630371	33	0.000	-1.336	2.1	gDNA	n.a.
chr08	q23.3 - q24.3	113972147	145986541	545	2.029	0.000	32.0	3	GenomePlex
chr08	q23.3 - q24.3	114387099	142694213	444	2.386	0.000	28.3	2	GenomePlex
chr08	q23.3 - q24.3	115354884	142694213	437	1.641	0.000	27.3	gDNA	n.a.
chr08	q23.3 - q24.3	115354884	142838160	439	2.536	0.000	27.5	1	GenomePlex
chr08	q23.3 - q24.3	115354884	142694213	437	1.182	0.000	27.3	5	Ampli1

Chromosome #	Aberration							Sample	Amplification method
	Cytoband	Start	Stop	# of probes	Amp	Del	size (Mb)	(# of cell/genomic DNA)	
chr08	q23.3 - q24.3	115354884	143280752	447	1.159	0.000	27.9	6	Ampli1
chr08	q23.3 - q24.3	115354884	142694213	437	1.326	0.000	27.3	8	Ampli1
chr08	q23.3 - q24.3	115354884	143333332	448	1.297	0.000	28.0	9	Ampli1
chr08	q23.3 - q24.3	115354884	142694213	437	1.340	0.000	27.3	10	Ampli1
chr08	q23.3 - q24.3	115499813	142694213	436	1.264	0.000	27.2	7	Ampli1
chr09	p24.3 - p21.1	1793603	30800291	450	0.000	-0.368	29.0	6	Ampli1
chr09	p21.3	21321182	21374442	4	3.846	0.000	0.1	7	Ampli1
chr09	p21.1 - p13.1	32446057	39146954	166	0.000	-0.469	6.7	7	Ampli1
chr09	p13.3 - p13.1	34075974	39146954	128	0.000	-0.447	5.1	10	Ampli1
chr09	p13.3 - p13.1	36090483	39146954	59	0.000	-0.557	3.1	gDNA	n.a.
chr09	q21.11 - q21.12	72395705	72744315	10	2.081	0.000	0.3	7	Ampli1
chr09	q32 - q34.3	114079223	140128736	632	0.000	-0.398	26.0	7	Ampli1
chr09	q33.3 - q34.3	129251335	140128736	306	0.366	0.000	10.9	9	Ampli1
chr10	p14	11690026	12111084	11	0.656	0.000	0.4	gDNA	n.a.
chr10	p12.2 - p11.21	23081808	34755676	226	0.439	0.000	11.7	7	Ampli1
chr10	q11.23 - q21.1	51736811	57140296	90	1.083	0.000	5.4	7	Ampli1
chr10	q21.3 - q26.11	68649437	119795433	1037	0.657	0.000	51.1	4	GenomePlex
chr10	q23.1 - q24.31	83868587	102695235	398	0.369	0.000	18.8	7	Ampli1
chr10	q23.33	95547420	95657839	4	3.315	0.000	0.1	7	Ampli1
chr11	p15.5 - p11.12	208365	51289318	970	0.341	0.000	51.1	gDNA	n.a.
chr11	p15.5 - p11.12	303837	50188486	965	0.458	0.000	49.9	4	GenomePlex
chr11	p15.3 - p12	10741917	42063359	534	0.318	0.000	31.3	7	Ampli1
chr11	q11 - q24.3	54866623	128399315	1663	0.488	0.000	73.5	3	GenomePlex
chr11	q11 - q25	54866623	133720992	1771	0.274	0.000	78.9	5	Ampli1
chr11	q11 - q25	55002923	134373617	1780	0.392	0.000	79.4	gDNA	n.a.
chr11	q11	56098684	56294673	4	3.188	0.000	0.2	7	Ampli1
chr11	q12.1 - q25	56850037	134160011	1747	0.391	0.000	77.3	9	Ampli1
chr11	q12.1 - q13.5	58983477	76572286	574	0.888	0.000	17.6	4	GenomePlex
chr11	q12.2 - q13.4	60634209	73593084	449	0.617	0.000	13.0	6	Ampli1
chr11	q12.2 - q13.4	60805584	72157759	406	0.728	0.000	11.4	9	Ampli1
chr11	q12.2 - q25	60926740	133781718	1625	0.304	0.000	72.9	8	Ampli1

Chromosome #	Aberration							Sample	Amplification method
	Cytoband	Start	Stop	# of probes	Amp	Del	size (Mb)	(# of cell/genomic DNA)	
chr11	q12.3 - q25	61443364	133720992	1602	0.725	0.000	72.3	2	GenomePlex
chr11	q12.3 - q25	62894164	133720992	1550	0.279	0.000	70.8	10	Ampli1
chr11	q13.4 - q24.3	71861247	130209175	1168	0.289	0.000	58.3	7	Ampli1
chr12	p12.2 - p11.1	21152676	34236852	252	0.351	0.000	13.1	gDNA	n.a.
chr12	p12.1 - p11.1	21422844	33713511	237	0.389	0.000	12.3	7	Ampli1
chr12	q12 - q24.33	43196437	132278059	1861	0.473	0.000	89.1	4	GenomePlex
chr12	q13.13 - q14.1	50214554	56396514	248	0.000	-0.382	6.2	7	Ampli1
chr12	q13.3 - q24.33	55348927	132031569	1478	0.599	0.000	76.7	1	GenomePlex
chr12	q14.1 - q23.2	56440283	101536818	743	0.000	-0.267	45.1	9	Ampli1
chr12	q14.1 - q23.2	56585353	100172938	696	0.000	-0.351	43.6	6	Ampli1
chr12	q23.2	100203668	101036489	26	1.575	0.000	0.8	7	Ampli1
chr12	q23.2	100901079	100973914	3	3.702	0.000	0.1	5	Ampli1
chr12	q24.11 - q24.33	107933407	131891124	527	0.000	-0.322	24.0	7	Ampli1
chr13	q11 - q13.3	18194544	37526008	408	0.339	0.000	19.3	gDNA	n.a.
chr13	q12.11 - q33.3	18424064	107032265	1566	0.000	-0.731	88.6	1	GenomePlex
chr13	q12.11 - q33.2	18679724	104320727	1525	0.522	0.000	85.6	7	Ampli1
chr13	q12.11 - q34	20358009	113532325	1660	0.000	-0.802	93.2	3	GenomePlex
chr13	q12.12	23330135	23467340	3	3.732	0.000	0.1	5	Ampli1
chr13	q12.12	23330135	23467340	3	3.875	0.000	0.1	7	Ampli1
chr13	q12.13 - q12.2	26518031	26766211	7	2.877	0.000	0.2	7	Ampli1
chr13	q22.1	72326167	73477139	26	1.798	0.000	1.2	7	Ampli1
chr14	q11.2 - q32.33	19323579	106311914	1730	0.000	-0.525	87.0	gDNA	n.a.
chr14	q11.2 - q32.33	19323579	106109254	1726	0.000	-0.410	86.8	5	Ampli1
chr14	q11.2 - q32.33	19408583	103376255	1658	0.000	-0.819	84.0	3	GenomePlex
chr14	q11.2 - q32.33	19796936	106329869	1723	0.000	-0.958	86.5	1	GenomePlex
chr14	q11.2 - q32.33	19869350	106109254	1713	0.000	-0.455	86.2	7	Ampli1
chr14	q11.2 - q32.33	19869350	103100647	1638	0.000	-0.375	83.2	8	Ampli1
chr14	q11.2 - q32.33	19918227	106160234	1712	0.000	-0.366	86.2	10	Ampli1
chr14	q11.2 - q32.2	20889193	99602032	1498	0.000	-0.348	78.7	9	Ampli1
chr14	q11.2 - q32.2	22722071	96018891	1390	0.000	-0.619	73.3	2	GenomePlex
chr14	q12 - q32.31	23837514	101801956	1448	0.000	-0.526	78.0	6	Ampli1

Chromosome #	Aberration							Sample	Amplification method
	Cytoband	Start	Stop	# of probes	Amp	Del	size (Mb)	(# of cell/genomic DNA)	
chr15	q11.1 - q26.3	18362555	100282878	1701	0.392	0.000	81.9	gDNA	n.a.
chr15	q11.1 - q26.3	18362555	100168718	1698	0.601	0.000	81.8	2	GenomePlex
chr15	q11.1 - q26.3	18362555	99831916	1689	0.345	0.000	81.5	5	Ampli1
chr15	q11.2 - q26.3	18741716	99831916	1688	0.329	0.000	81.1	6	Ampli1
chr15	q11.2 - q26.3	18741716	99831916	1688	0.393	0.000	81.1	9	Ampli1
chr15	q11.2 - q26.3	18835660	100282878	1699	0.413	0.000	81.4	7	Ampli1
chr15	q11.2 - q26.3	18863056	99999936	1689	0.348	0.000	81.1	8	Ampli1
chr15	q11.2 - q26.3	18863056	100282878	1698	0.335	0.000	81.4	10	Ampli1
chr15	q14	33837136	35854510	28	1.641	0.000	2.0	7	Ampli1
chr15	q15.3	42580170	42642265	3	4.794	0.000	0.1	5	Ampli1
chr15	q15.3	42580170	42642265	3	5.285	0.000	0.1	6	Ampli1
chr15	q15.3 - q21.1	42580170	42797075	10	3.446	0.000	0.2	7	Ampli1
chr15	q21.1	45459710	45688119	6	2.987	0.000	0.2	7	Ampli1
chr15	q21.3 - q25.1	53700142	77395078	562	0.647	0.000	23.7	4	GenomePlex
chr15	q21.3	54108233	55070905	27	1.733	0.000	1.0	7	Ampli1
chr15	q26.1	87238297	87256705	3	0.000	-2.857	0.0	10	Ampli1
chr16	p13.3 - p11.2	712555	34129822	744	0.653	0.000	33.4	4	GenomePlex
chr16	p13.2	6328649	6725667	12	0.000	-2.507	0.4	gDNA	n.a.
chr16	p13.2	6328649	6725667	12	0.000	-2.387	0.4	8	Ampli1
chr16	p13.2	6367978	6725667	10	0.000	-2.964	0.4	7	Ampli1
chr16	p13.2	6367978	6725667	10	0.000	-2.555	0.4	10	Ampli1
chr16	p13.2	6406832	6492945	3	0.000	-5.517	0.1	gDNA	n.a.
chr16	p13.2	6492886	6725667	6	0.000	-3.620	0.2	5	Ampli1
chr16	p13.2	6492886	6725667	6	0.000	-3.613	0.2	6	Ampli1
chr16	p13.2	6492886	6725667	6	0.000	-3.465	0.2	9	Ampli1
chr16	p13.2	6644617	6725667	4	0.000	-4.572	0.1	gDNA	n.a.
chr16	p13.2	6821092	7322964	10	0.000	-0.644	0.5	gDNA	n.a.
chr16	p12.3	19426488	19547492	5	2.696	0.000	0.1	7	Ampli1
chr16	p12.1 - p11.1	24529935	34903000	208	0.000	-0.402	10.4	7	Ampli1
chr16	q11.2 - q24.3	45174916	88621982	997	0.590	0.000	43.4	4	GenomePlex
chr16	q12.1 - q12.2	51072014	52376121	27	1.365	0.000	1.3	7	Ampli1

Chromosome #	Aberration							Sample	Amplification method
	Cytoband	Start	Stop	# of probes	Amp	Del	size (Mb)	(# of cell/genomic DNA)	
chr16	q13 - q24.3	55157063	88638764	803	0.276	0.000	33.5	9	Ampli1
chr16	q21	57219863	58987235	23	1.280	0.000	1.8	5	Ampli1
chr16	q21	57785182	58987235	13	2.773	0.000	1.2	7	Ampli1
chr16	q23.1	77613911	77745538	4	0.000	-1.463	0.1	gDNA	n.a.
chr17	p13.3 - p11.1	87009	22129948	601	0.000	-0.423	22.0	5	Ampli1
chr17	p13.3 - p11.1	87009	22129948	601	0.000	-0.425	22.0	8	Ampli1
chr17	p13.3 - p11.2	87009	21386319	596	0.000	-0.750	21.3	7	Ampli1
chr17	p13.3 - p11.1	148092	22129948	600	0.000	-0.435	22.0	gDNA	n.a.
chr17	p13.3 - p11.2	756888	21857788	586	0.000	-0.543	21.1	10	Ampli1
chr17	p13.3 - p11.2	1315406	19866433	540	0.642	0.000	18.6	4	GenomePlex
chr17	q11.1 - q25.3	23134547	78623230	1540	0.717	0.000	55.5	4	GenomePlex
chr17	q11.2 - q25.3	23393985	77804425	1504	0.288	0.000	54.4	9	Ampli1
chr17	q11.2 - q12	26284942	34360657	193	0.653	0.000	8.1	gDNA	n.a.
chr17	q11.2 - q21.2	26284942	36979959	300	0.414	0.000	10.7	5	Ampli1
chr17	q11.2 - q21.2	26284942	37217631	309	0.364	0.000	10.9	8	Ampli1
chr17	q11.2 - q21.32	26284942	43404016	533	0.424	0.000	17.1	6	Ampli1
chr17	q12	29637473	34188999	108	0.520	0.000	4.6	10	Ampli1
chr17	q12	33121121	34031410	10	1.310	0.000	0.9	gDNA	n.a.
chr17	q21.2 - q22	37134361	53773585	458	0.000	-0.301	16.6	7	Ampli1
chr17	q24.1 - q24.3	60370406	66340623	124	0.611	0.000	6.0	7	Ampli1
chr17	q24.1 - q24.2	60744623	63719686	62	0.752	0.000	3.0	gDNA	n.a.
chr17	q24.3	66210775	68242871	24	0.000	-0.553	2.0	gDNA	n.a.
chr17	q24.3 - q25.3	66421144	78300467	349	0.000	-0.308	11.9	7	Ampli1
chr18	p11.32 - p11.21	4316	14148122	270	0.422	0.000	14.1	gDNA	n.a.
chr18	p11.32 - p11.21	132096	14723870	273	0.336	0.000	14.6	10	Ampli1
chr18	p11.32 - p11.21	2276141	13656290	221	0.500	0.000	11.4	7	Ampli1
chr18	q11.1 - q23	16793851	76083117	968	0.000	-0.479	59.3	7	Ampli1
chr18	q11.1 - q23	16793851	76083117	968	0.000	-0.393	59.3	8	Ampli1
chr18	q11.1 - q22.3	16874053	70846966	880	0.000	-0.793	54.0	3	GenomePlex
chr18	q11.1 - q23	16874053	75994936	965	0.000	-0.492	59.1	5	Ampli1
chr18	q11.1 - q23	16938683	73758157	923	0.000	-0.457	56.8	9	Ampli1

Chromosome #	Aberration							Sample	Amplification method
	Cytoband	Start	Stop	# of probes	Amp	Del	size (Mb)	(# of cell/genomic DNA)	
chr18	q11.1 - q23	16976046	75863450	960	0.000	-0.659	58.9	6	Ampli1
chr18	q11.1 - q23	17030723	75642167	953	0.000	-1.067	58.6	1	GenomePlex
chr18	q11.2 - q23	17311108	75994936	954	0.000	-0.372	58.7	10	Ampli1
chr18	q11.2 - q23	17520584	76018409	949	0.000	-0.608	58.5	gDNA	n.a.
chr18	q11.2 - q23	17520584	75004861	926	0.000	-0.777	57.5	2	GenomePlex
chr19	p13.3 - p12	278273	24132581	873	0.000	-0.348	23.9	gDNA	n.a.
chr19	p13.3 - p12	278273	22511226	849	0.000	-0.401	22.2	8	Ampli1
chr19	p13.3 - p12	278273	22303125	845	0.000	-0.546	22.0	10	Ampli1
chr19	p13.3 - p13.2	737550	10656335	378	1.021	0.000	9.9	4	GenomePlex
chr19	p13.3 - p12	774554	21768496	825	0.000	-0.810	21.0	7	Ampli1
chr19	p13.3 - p12	1428461	21880489	799	0.000	-0.394	20.5	5	Ampli1
chr19	q13.11 - q13.43	37780053	63161649	1030	0.327	0.000	25.4	5	Ampli1
chr19	q13.11 - q13.43	37985252	63784382	1062	0.296	0.000	25.8	8	Ampli1
chr19	q13.11 - q13.43	38106196	63784382	1057	0.550	0.000	25.7	gDNA	n.a.
chr19	q13.11 - q13.43	38131058	63784382	1055	0.604	0.000	25.7	6	Ampli1
chr19	q13.11 - q13.43	38131058	63784382	1055	0.658	0.000	25.7	9	Ampli1
chr19	q13.11 - q13.43	38156265	63342285	1024	0.922	0.000	25.2	2	GenomePlex
chr19	q13.2 - q13.33	43433697	56229403	544	0.930	0.000	12.8	4	GenomePlex
chr20	p12.1	14449723	15823849	27	0.000	-1.189	1.4	gDNA	n.a.
chr20	p12.1	14939493	15046492	3	0.000	-2.626	0.1	8	Ampli1
chr20	p12.1	14939493	15046492	3	0.000	-3.034	0.1	10	Ampli1
chr20	p11.21 - p11.1	25227228	26135794	17	0.702	0.000	0.9	gDNA	n.a.
chr20	q11.21 - q13.33	29352138	62363633	757	0.728	0.000	33.0	gDNA	n.a.
chr20	q11.21 - q13.33	29352138	62363633	757	0.716	0.000	33.0	6	Ampli1
chr20	q11.21 - q13.33	29352138	62134908	745	0.488	0.000	32.8	8	Ampli1
chr20	q11.21 - q13.33	29352138	62363633	757	0.713	0.000	33.0	9	Ampli1
chr20	q11.21 - q13.12	29440414	44181278	368	0.800	0.000	14.7	4	GenomePlex
chr20	q11.21 - q13.33	29454882	61785825	727	1.182	0.000	32.3	2	GenomePlex
chr20	q11.21 - q13.33	29657275	62194881	741	0.860	0.000	32.5	3	GenomePlex
chr20	q11.21 - q13.33	29692858	62213560	742	0.573	0.000	32.5	5	Ampli1
chr20	q11.21 - q13.33	29977659	59035629	633	0.416	0.000	29.1	7	Ampli1

Chromosome #	Aberration							Sample (# of cell/genomic DNA)	Amplification method
	Cytoband	Start	Stop	# of probes	Amp	Del	size (Mb)		
chr20	q11.21 - q13.33	29997987	59035629	632	0.432	0.000	29.0	10	Ampli1
chr20	q11.22 - q13.33	32101066	62363633	685	0.865	0.000	30.3	1	GenomePlex
chr21	q11.2 - q22.11	13562263	34477354	391	0.000	-0.465	20.9	9	Ampli1
chr21	q11.2 - q22.3	13956118	46811587	715	0.000	-0.382	32.9	8	Ampli1
chr21	q11.2 - q22.3	14092232	46892352	719	0.000	-0.574	32.8	gDNA	n.a.
chr21	q11.2 - q22.3	14372903	46801599	710	0.000	-0.424	32.4	10	Ampli1
chr21	q11.2 - q22.3	14460851	46223019	685	0.000	-0.992	31.8	1	GenomePlex
chr21	q11.2 - q22.3	14876312	46880878	703	0.000	-0.324	32.0	6	Ampli1
chr21	q21.1 - q22.3	18499175	46488180	617	0.000	-0.325	28.0	5	Ampli1
chr21	q22.11 - q22.3	31374122	46801599	409	0.000	-0.451	15.4	7	Ampli1
chr22	q11.1 - q11.21	15476855	17021468	34	0.000	-0.431	1.5	gDNA	n.a.
chr22	q11.21	17299942	19712953	74	0.882	0.000	2.4	9	Ampli1
chr22	q11.21	17364460	19605314	64	0.736	0.000	2.2	gDNA	n.a.
chr22	q11.21	17403824	19432006	59	0.897	0.000	2.0	5	Ampli1
chr22	q11.21	17809644	19125851	36	0.843	0.000	1.3	8	Ampli1
chr22	q11.21	17964758	19432006	36	1.516	0.000	1.5	6	Ampli1
chr22	q11.21 - q13.33	19653161	49525130	799	0.000	-0.681	29.9	7	Ampli1
chr22	q11.21 - q13.33	19674757	49525130	798	0.000	-0.380	29.9	gDNA	n.a.
chr22	q11.21 - q13.33	19674757	49034395	781	0.000	-0.460	29.4	10	Ampli1
chr22	q12.1 - q12.2	26828859	28611320	54	0.482	0.000	1.8	gDNA	n.a.
chr22	q12.1 - q12.2	26839313	28611320	53	0.295	0.000	1.8	7	Ampli1
chr22	q12.1 - q12.2	26839313	28683083	54	0.384	0.000	1.8	10	Ampli1
chr22	q12.2 - q13.32	28683025	47672822	519	0.000	-0.418	19.0	8	Ampli1
chr22	q12.2 - q13.33	28683025	49034395	552	0.000	-0.423	20.4	5	Ampli1

Supplementary Tab. S4. Details of gains and losses (Mb) detected in single HT-29 cells (array-CGH) – sorted according to location within genome and cell

Chromosome #	GenomePlex-based amplification				Ampli1-based amplification					
	Non-C&R treatment		C&R treatment		Non-C&R treatment			C&R treatment		
	#1	#2	#3	#4	#5	#6	#7	#8	#9	#10
1				103.54			44.40			15.00
				91.31			5.09			
				0.43			0.34			
							0.09			
							0.003			
2							79.08		0.09	
							12.81			
							3.93			
							0.57			
							0.50			
							0.26			
							0.15			
3	89.43	88.69	89.06		89.23	89.73	98.31	88.46	89.99	86.83
					0.71	0.34	52.09	0.34	31.19	81.23
					0.34		15.96		0.34	0.34
							7.45			
							0.84			
							0.55			
							0.40			
							0.24			
							0.15			
4					9.66	105.56	9.38	31.62	32.40	30.02
							2.19			
							0.87			
							0.65			
							0.26			
							0.15			
							0.11			
							0.05			
5				39.49	41.46		38.40	44.91		44.54
							1.10			
							0.96			
							0.05			
6	104.94			7.81	19.20	57.48	43.11	22.98	22.27	22.76
					14.01	21.59	11.40		21.05	
							10.31			
							6.37			
							1.75			
							1.12			
							0.39			
							0.17			
7	95.82		4.18		56.77	35.57	72.92		29.75	
					0.47		50.49			

Chromosome #	GenomePlex-based amplification				Ampli1-based amplification					
	Non-C&R treatment		C&R treatment		Non-C&R treatment			C&R treatment		
	#1	#2	#3	#4	#5	#6	#7	#8	#9	#10
7							10.15			
							2.68			
							1.14			
							0.47			
							0.35			
							0.34			
							0.20			
							0.16			
							0.12			
							0.05			
8	43.08	43.17	43.24	91.39	43.17	58.87	43.24	43.24	42.47	43.24
	27.48	28.31	32.01		27.34	43.02	27.19	27.34	27.98	27.34
					0.08	27.93	0.26	0.08	0.08	
						0.08	0.21			
							0.08			
9						29.01	26.05		10.88	5.07
							6.70			
							0.35			
							0.05			
10				51.15			18.83			
							11.67			
							5.40			
							0.11			
11		72.28	73.53	49.88	78.85	12.96	58.35	72.85	77.31	70.83
				17.59			31.32		11.35	
							0.20			
12	76.68			89.08	0.07	43.59	23.96		45.10	
							12.29			
							6.18			
							0.83			
13	88.61		93.17		0.14		85.64			
							1.15			
							0.25			
							0.14			
14	86.53	73.30	83.97		86.79	77.96	86.24	83.23	78.71	86.24
15		81.81		23.69	81.47	81.09	81.45	81.14	81.09	81.42
					0.06	0.06	2.02			0.02
							0.96			
							0.23			
							0.22			
16				43.45	1.77	0.23	10.37	0.40	33.48	0.36
				33.42	0.23		1.30		0.23	
							1.20			
							0.36			
							0.12			
17				55.49	22.04	17.12	21.30	22.04	54.41	21.10

Chromosome #	GenomePlex-based amplification				Ampli1-based amplification					
	Non-C&R treatment		C&R treatment		Non-C&R treatment			C&R treatment		
	#1	#2	#3	#4	#5	#6	#7	#8	#9	#10
17				18.55	10.70		16.64	10.93		4.55
							11.88			
							5.97			
18	58.61	57.48	53.97		59.12	58.89	59.29	59.29	56.82	58.68
							11.38			14.59
19		25.19		12.80	25.38	25.65	20.99	25.80	25.65	22.02
				9.92	20.45			22.23		
20	30.26	32.33	32.54	14.74	32.52	33.01	29.06	32.78	33.01	29.04
								0.11		0.11
21	31.76				27.99	32.00	15.43	32.86	20.92	32.43
22					20.35	1.47	29.87	18.99	2.41	29.36
					2.03		1.77	1.32		1.84

Supplementary Table S5. Ampli1 CHPCustom Beta panel target gene list

Chromosomes	Genes	Amplicon #	Hotspot #
chr9	*ABL1*	5	19
chr2	*ALK*	1	1
chr11	*ATM*	9	13
chr16	*CDH1*	3	4
chr5	*CSF1R*	2	8
chr7	*EGFR*	7	71
chr2	*ERBB4*	4	7
chr4	*FBXW7*	3	23
chr10	*FGFR2*	3	5
chr13	*FLT3*	4	30
chr20	*GNAS*	2	Dataset12
chr2	*IDH1*	1	15
chr19	*JAK3*	3	6
chr4	*KIT*	6	114
chr7	*MET*	3	6
chr1	*MPL*	1	10
chr1	*NRAS*	3	35
chr3	*PIK3CA*	5	60
chr12	*PTPN11*	2	28
chr10	*RET*	2	4
chr22	*SMARCB1*	3	10
chr2	*SRC*	1	1
chr17	*TP53*	8	1147
chr14	*AKT1*	2	6
chr5	*APC*	2	17
chr7	*BRAF*	2	77
chr9	*CDKN2A*	2	97
chr3	*CTNNB1*	1	73
chr17	*ERBB2*	4	19
chr7	*EZH2*	1	11
chr8	*FGFR1*	2	2
chr4	*FGFR3*	5	17
chr19	*GNA11*	1	5
chr12	*HNF1A*	2	10
chr15	*IDH2*	1	12
chr4	*KDR*	6	8
chr12	*KRAS*	3	63
chr3	*MLH1*	1	1
chr9	*NOTCH1*	3	20
chr4	*PDGFRA*	4	26
chr10	*PTEN*	2	55
chr13	*RB1*	4	9
chr18	*SMAD4*	4	13
chr7	*SMO*	4	4
chr19	*STK11*	4	23
chr3	*HVHL*	2	68
